# Role of DFNB1 mutations in hereditary hearing loss among assortative mating hearing impaired families from South India

**DOI:** 10.1186/s12881-018-0609-6

**Published:** 2018-06-19

**Authors:** Pavithra Amritkumar, Justin Margret Jeffrey, Jayasankaran Chandru, Paridhy Vanniya S, M. Kalaimathi, Rajagopalan Ramakrishnan, N. P. Karthikeyen, C. R. Srikumari Srisailapathy

**Affiliations:** 1 Department of Genetics, Dr. ALM Post Graduate Institute of Basic Medical Sciences, University of Madras, Taramani, Chennai, 600113 India; 2 Current affiliation: PG and Research Department of Biotechnology, Women’s Christian College, Chennai, India; 3 Department of ENT, SRM Medical College Hospital and Research Centre, SRM Institute of Science and Technology, Kattankulathur, India; 4 DOAST Hearing Care Center, Anna Nagar, Chennai, 600040 India

**Keywords:** Assortative mating, *GJB2* mutations, *GJB6* mutations, DFNB1, Deafness, South India

## Abstract

**Background:**

DFNB1, the first locus to have been associated with deafness, has two major genes *GJB2* & *GJB6,* whose mutations have played vital role in hearing impairment across many ethnicities in the world. In our present study we have focused on the role of these mutations in assortative mating hearing impaired families from south India.

**Methods:**

One hundred and six assortatively mating hearing impaired (HI) families of south Indian origin comprising of two subsets: 60 deaf marrying deaf (DXD) families and 46 deaf marrying normal hearing (DXN) families were recruited for this study. In the 60 DXD families, 335 members comprising of 118 HI mates, 63 other HI members and 154 normal hearing members and in the 46 DXN families, 281 members comprising of 46 HI and their 43 normal hearing partners, 50 other HI members and 142 normal hearing family members, participated in the molecular study. One hundred and sixty five (165) healthy normal hearing volunteers were recruited as controls for this study. All the participating members were screened for variants in *GJB2* and *GJB6* genes and the outcome of gene mutations were compared in the subsequent generation in begetting deaf offspring.

**Results:**

The DFNB1 allele frequencies for DXD mates and their offspring were 36.98 and 38.67%, respectively and for the DXN mates and their offspring were 22.84 and 24.38%, respectively. There was a 4.6% increase in the subsequent generation in the DXD families, while a 6.75% increase in the DXN families, which demonstrates the role of assortative mating along with consanguinity in the increase of DFNB1 mutations in consecutive generations. Four novel variants, p.E42D (in *GJB2* gene), p.Q57R, p.E101Q, p.R104H (in *GJB6* gene) were also identified in this study.

**Conclusion:**

This is the first study from an Indian subcontinent reporting novel variants in the coding region of *GJB6* gene. This is perhaps the first study in the world to test real-time, the hypothesis proposed by Nance et al. in 2000 (intense phenotypic assortative mating mechanism can double the frequency of the commonest forms of recessive deafness [DFNB1]) in assortative mating HI parental generation and their offspring.

## Background

Hearing is one of the vital sensations, which keeps humans connected with each other and the world around. Consequently, hearing loss can have a profound impact on cognitive, psychosocial and educational development of an individual. Greater part of our present day knowledge on the physiology of hearing has come from various studies on hearing loss.

Till date, nearly 80 genes in over 142 deafness loci are associated with non-syndromic hearing loss (NSHL) reflecting the heterogenic and complex nature of the mechanism of hearing. Approximately 1200 different deafness-causing mutations are identified across the human genome. However, mutations do not occur at same frequencies across ethnicities. Eleven autosomal recessive loci (DFNB1, DFNB3, DFNB4, DFNB5, DFNB6, DFNB7/11, DFNB12, DFNB15, DFNB17, DFNB18 and DFNB95) and one autosomal dominant locus (DFNA59) are currently known to be associated with hearing loss in India (Hereditary hearing loss homepage, http://hereditaryhearingloss.org/). Despite this genetic heterogeneity across ethnicities, DFNB1 locus on chromosome 13q11–12, accounts for up to 50% of NSHL [1, 2]. The first deafness associated gene in the DFNB1 locus, the *GJB2* gene (GenBank M86849, MIM 121011) coding the gap junction protein, Connexin 26 (Cx26), was reported in 1997 [3]. Connexin 26 protein is found in the cochlea of the inner ear and is a major regulator of K^+^ homeostasis. In the absence of K^+^ circulation, the hair cells are unable to generate action potential in response to sound. Recent studies have suggested that they play an important role in inter and intracellular signaling pathways of the inner ear [4]. Over 220 mutations, polymorphisms and unknown variants in the *GJB2* gene have been reported worldwide ([5]; Connexins and deafness homepage, http://davinci.crg.es/deafness/).High prevalence of *GJB2* mutations among many populations has made it necessary to depend on molecular testing for diagnosis. However, nearly 10–50% of deaf subjects in many studies showing only one *GJB2* mutant allele, further complicated the molecular diagnosis of DFNB1 deafness [6]. This led to the hypothesis that there could be other mutations in the DFNB1 locus but outside the *GJB2* gene. Subsequently, two large deletions occurring in the *GJB6* gene, which encodes connexin 30 (Cx30) protein and lying ~ 35 kb telomeric to *GJB2* on chromosome 13, were reported [6, 7]. Cx30 protein is of size 30 kDa, having 261 amino acids and shares 77% identity with Cx26. Cx26/Cx30 cochlear gap junctions forming heteromeric channels have been implicated in the maintenance of K+ homeostasis in the inner ear and contributing to the inner ear homeostasis [8, 9]. The *GJB6* gene was first described as a causative in a rare dominant form of deafness, DFNA3, and its implication in NSHL were ascertained through the identification of two large deletions, del(GJB6-D13S1830) of size 309 kb and del(GJB6-D13S1854) of size 232 kb, which truncate the *GJB6* gene [6, 7]. Till date, only four point mutations and four deletions in the *GJB6* gene or the region upstream have been reported (http://hereditaryhearingloss.org/). Studies on common mutations in assortative mating families have not been accomplished in the Indian subcontinent till date, except for our preliminary findings from this study [10–12].

In the hearing impaired (HI) population, assortative mating refers to the preference of a HI individual to marry another HI individual (deaf marrying deaf, or DXD) or a HI individual opting for a normal hearing individual as a partner (deaf marrying normal hearing, or DXN), with hearing impairment forming the basis for selection or non-selection. Segregation analysis with respect to the distribution of deaf and hearing offspring in such mating scan provide estimation of the proportion of such marriages that can have only deaf children (non-complementary matings), only hearing children (complementary matings), and those capable of producing both deaf and hearing children. A non-complementary mating is when both the deaf mates are homozygous for recessive alleles at the same locus, and can therefore produce only deaf offspring, while a complementary mating could be when mates either have non-genetic deafness or a combination of non-genetic deafness and recessive deafness, or both the mates having different forms of recessive deafness [13–15].

There are very few reports available on the mutational dynamics of assortative mating among the HI. The available reports state that between nineteenth and twentieth centuries, the frequency of HI children in the US with one or two HI parents increased by 38% from 0.064 to 0.089 [15]. These reports have focused only on assortative mating HI families as consanguinity as a practice was absent in these regions. Consanguineous marriage is a tradition that is commonly practiced among many parts of the world especially in North and Sub-Saharan Africa, Latin American communities, West, Central and South Asia where there is 10–50% prevalence of consanguinity among their general population [16]. In India, especially Tamil Nadu, Andhra Pradesh, and Karnataka overwhelmingly prefer and practice consanguineous marriages across all major religious groups and ethnic entities. There have been studies on hereditary hearing loss from south India reporting parental consanguinity of deaf subjects screened ranging between 32.55 and 54.10% [17–19] In Tamil Nadu, the studies on childhood hearing impaired have shown parental consanguinity ranging from 28 to 50% [20].

Consanguinity leads to an increase in identity by decent for all loci, indiscriminately. In contrast, once recessive genes are expressed phenotypically, assortative mating creates “gametic phase disequilibrium” [21], or the non-random association and gametic transmission of potentially very rare alleles at unlinked loci (genocopies) that have similar effects on the phenotype. Thus genetic heterogeneity and consanguinity add further complexity to the genetic studies on assortative mating deaf families. There are no genetic studies till date that have addressed the genetic and socio demographic dynamics simultaneously, on assortatively mating HI families from India.

Deaf marrying deaf is an increasing trend with sign language being the preferred mode of communication. Despite their preferential choice of a HI mate, the preferential desire for offspring’s hearing status has largely been only normal hearing. The HI mates consider their deafness as a disability, which is a sharp deviation from ‘Deaf culture’ prevalent in the western HI population. With more than 50% of hearing loss having genetic predisposition, it is very important to systematically analyze the role of genetic mutations in such matings. Therefore, screening for common mutations associated with hearing loss among assortatively mating HI couples and their families in Indian population would be essential to understand the role of incidence of hearing impairment in the subsequent generation, which forms the basis for this study.

## Methods

### Recruitment of participants and clinical data collection

One hundred and six (106) assortatively mating hearing impaired families comprising of **60 deaf marrying deaf, or DXD families** and **46 deaf marrying normal hearing, or DXN families**, predominantly from south India, with no familial interconnectivity, were recruited for this study. All international standards for ethical research were met and the Institutional Human Ethical Committee of the University of Madras, Post Graduate Institute of Basic Medical Sciences, Chennai, India, approved the study (Ref Nos: PGIBMS/CO/Human Ethical/2010–11/1458, PGIBMS/CO/Human Ethical/2011–12/546, IHEC Approval No: UM/IHEC/11–2013-I). Assortatively mating hearing impaired (HI) families were primarily identified through adult deaf organizations and associations for the HI, Alumni and Parent- Teacher associations of deaf schools in the four states (Andhra Pradesh, Karnataka, Kerala and Tamil Nadu) of south India. Some families were also referred by ENT surgeons, audiologists, gynecologists and neonatologists wherein the family members were seeking genetic counseling pertaining to the incidence of hearing loss in the family. Only those families in which the proband was prelingual HI and married to a partner who was either of normal hearing status (DXN) or was also prelingual HI (DXD), with at least two generations of family members available for the study, were included. Written informed consent was obtained from all participants in every family. Detailed family pedigrees were drawn. Information on demography, nativity, consanguinity, age at onset of hearing loss, detailed prenatal and perinatal history, use of ototoxic drugs (aminoglycosides), etc. was documented through a structured schedule. Attitudinal preferences of the HI mates as well as the hearing partner in each of the family towards choice of mate, parental choice, preference towards hearing status of their child/ children, mode of communication and genetic testing were also documented. Where both the mates were HI, information was obtained from at least two speaking relatives well informed about the family. Pre-test genetic counseling was provided to each of these families with the help of a sign language expert in our team. The degree of hearing loss for the participating members was evaluated through pure tone audiometry by measuring the air and bone conduction thresholds.

A total of 621 members comprising of hearing impaired and normal hearing from these 106 assortative mating families were recruited for this study (Table 1).

**Table 1:** Distribution of hearing impaired and normal hearing members in the assortative mating families

Type of Mating	No. of hearing impaired mates	No. of hearing partners	Other hearing impaired members in the family	Other hearing members in the family	Total
Deaf marrying deaf (DXD)	120	0	63	154	337
Deaf marrying normal hearing (DXN)	46	46	50	142	284
TOTAL	166	46	113	296	621

### *GJB2* and *GJB6* mutation analysis

Genomic DNA was extracted by standard PCI method [22]. The coding region of GJB2 gene (exon 2) was PCR-amplified using primer pair GJB2-EX2-1F (5′ -TCT CCC TGT TCT GTC CTA GC-30) and GJB2-EX2-1R (50-GAC AGC ATG AGA GGG ATG AG-3′) with annealing temperature of 62 °C. Amplification of the non-coding first exon and the flanking donor splicing site was carried out using Advantage-GC Genomic PCR kit (Clontech, Mountain View, USA) and PCR primers EXON 1A (5’-TCC GTA ACT TTC CCA GTC TCC GAG GGA AGA GG-3′) and EXON 1 M (5′ -CCC AAG GAC GTG TGT TGG TCC AGC CCC-3′) with conditions previously described by Ramshanker et al. [17], for all the HI members. Coding exon (exon 6) of *GJB6* gene was amplified by hot-start PCR using overlapping primer pairs: GJB6-1F (5′- AGA CTA GCA GGG CAG GGA GT- 3′) and GJB6-1R (5′- AGG GGT CAA TCC CAC ATT TC -3′) measuring 676 bp; GJB6-2F (5′ -GAT AGA GGG GTC GCT GTG GT -3′) and GJB6-2R (5′- GGC TAC AGA AGG AAC TTT CAG G -3′) measuring 494 bp with annealing temperature of 63 °C for both.

The amplified products were purified using QIAquick® PCR purification kit (Qiagen, Valencia, CA, USA). Bidirectional sequencing of the purified PCR products were carried out applying the same set of primers and ABI Prism Big-Dye Terminator 3.1 cycle sequencing reaction kit on an ABI 3730 automated sequencer (Applied Biosystems, Foster City, USA). The chromatogram sequences obtained were compared with the reference sequences of *GJB2* and *GJB6* in National Center for Biotechnology Information (NCBI: http://www.ncbi.nlm.nih.gov/) to identify any nucleotide base-pair changes.

Additionally, the affected members were screened for the presence of two large deletions in the *GJB6* gene, del (GJB6-D13S1830) and del (GJB6-D13S1854) by amplifying the regions containing the breakpoint fragments [6, 7].

### In silico analysis of novel variants

The bioinformatics tools used in this study to analyze the novel variants observed in the *GJB2* and *GJB6* genes were (i) Sorting Intolerant From Tolerant (SIFT), a sequence homology-based tool that predicts the phenotypic effect of amino acid substitution in a protein by scoring the substitution as tolerant or intolerant on the basis of sequence homology and physical properties of amino acids [23] and (ii) PolyPhen (Polymorphism Phenotyping), a tool which predicts possible impact of an amino acid substitution on the structure and function of a human protein using straight forward physical and comparative considerations [24]. The amino acid sequences of the native and variant proteins were then individually analyzed for physicochemical characteristics by Expasy’s online ProtParam tool available at http://web.expasy.org/protparam/ [25], and the results were compared.

### Homology modeling

The three dimensional structure of human *GJB6* was modeled from its protein sequence using the *automatic modelling mode* SWISS MODEL repository (http://swissmodel.expasy.org/). This resultant model was based on the template 2zw3 (*GJB2*), which shared 74.42% sequence identity with *GJB6*. It should be noted that the modeled residue range for *GJB6* was from amino acid 2 to 216 for a single chain. The protein structures were then minimized energetically using Swiss-PdbViewer [26]. The energy of the minimized protein was recorded. The native model was then mutated at the specified amino acid position using the “mutate” option in Swiss-PdbViewer, energy-minimized and the layer was saved as a “.pdb” file. The native and mutated proteins were crosschecked for alterations using Ramachandran plot at RAMPAGE portal available at http://mordred.bioc.cam.ac.uk/~rapper/rampage.php [27].

### Control study

One hundred and sixty five (165) healthy and normal hearing volunteers aged 19 to 66 years, belonging to different castes and states of south India were recruited as controls for this study and were screened for the most common variants in *GJB2* and *GJB6* genes. All the controls were subjected to audiological profiling to record their normal hearing status.

## Results

In the present study, consanguinity was recorded at two levels, parental consanguinity of the hearing impaired partners and consanguinity among the assortative mating partners in the 106 HI families. It was observed that consanguinity was conspicuously high among the normal hearing parents of the 120 DXD mates (45%), compared to that observed in the DXD mating (3.33%) (Table 2). Parental consanguinity of affected partners of 46 DXN mating was lower (32.61%) than the consanguinity observed in the DXN mating (39.13%) (Table 3). Additionally, the parental consanguinity of normal hearing mates in DXN mating (10.87%) was comparable with the parental consanguinity of the control group (11.51%), both reflecting the consanguinity trend in the general population.

**Table 2:** Consanguinity in parents of DXD mating and in DXD mating

Type of marriage based on consanguinity	PARENTAL CONSANGUINITY			In DXD mating
	In husbands’ parents (%)	In wives’ parents (%)	Combined (%)	
Consanguineous	24 (40%)	30 (50%)	54 (45%)	2 (3.33%)
Non Consanguineous	36 (60%)	30 (50%)	66 (55%)	58 (96.67%)
TOTAL	60	60	120	60

**Table 3:** Consanguinity in parents of DXN mating and in DXN mating

Type of marriage based on consanguinity	PARENTAL CONSANGUINITY			In DXN mating
	In husbands’ parents (%)	In wives’ parents (%)	Combined (%)	
Consanguineous	17 (36.96%)	13 (28.26%)	30 (32.61%)	18 (39.13%)
Non Consanguineous	29 (63.04%)	33 (71.74%)	62 (67.39%)	28 (60.87%)
TOTAL	46	46	92	46

In the first subgroup of 60 DXD families, 335 members comprising of 118 HI mates, 63 other HI members and 154 normal hearing members participated in the molecular study. In the second subgroup of 46 DXN families, 281 members comprising of 46 HI and their 43 normal hearing partners, 50 other HI members and 142 normal hearing family members, participated in the molecular study. Two HI mates in DXD families and three normal hearing partners in DXN families did not consent for blood sampling.

### Outcome of *GJB2* mutation screening

**Fig. 1:**
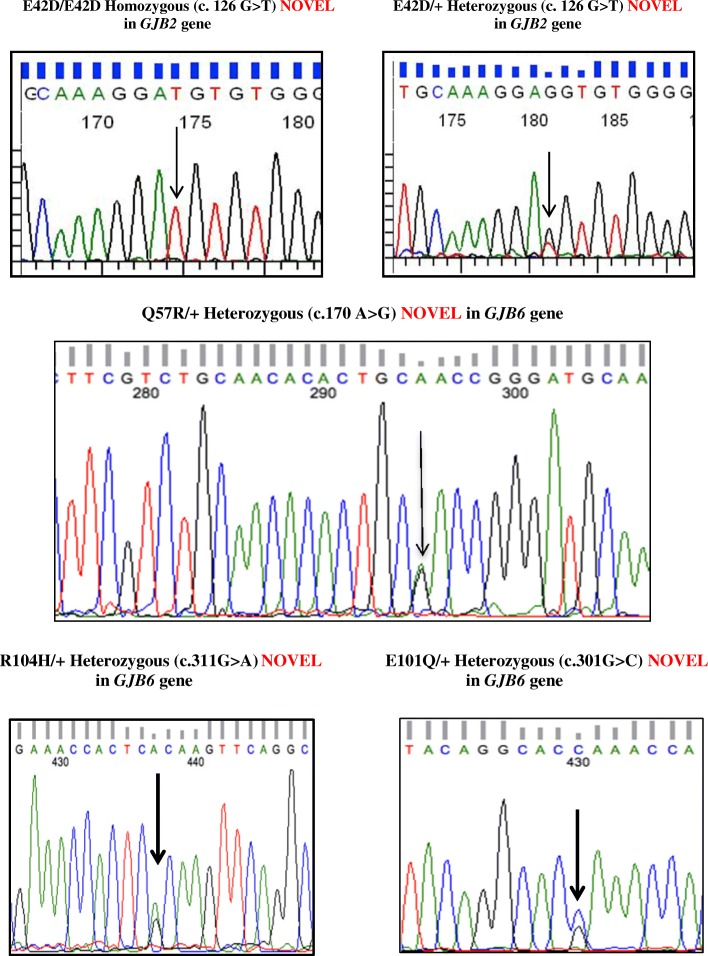
Partial chromatograms of *GJB2* and *GJB6* variants observed in the study

Twenty three *GJB2* variants were observed; 11 pathogenic, one novel variant and 11 polymorphisms (Table 4). Out of the 118 HI mates, 63.79% (37/58) of the deaf husbands and 66.67% (40/60) of the deaf wives had at least one nucleotide change in the *GJB2* gene. Fifteen different mutations/ variants in the *GJB2* gene were observed among the 118 HI mates (Table 5). **A novel mutation, p.E42D** hitherto unreported was observed in this study (Fig. 1).

**Table 4:** Summary of mutations/ variants in *GJB2* gene observed among the DXD, DXN mates and normal hearing controls

S. No.	GJB2 VARIANTS		DOMAIN/ LOCATION	No. of alleles in DXD mates (n=236)	Frequency in DXD mating (%)	No of alleles in affected partners of DXN mating (n=92)	Frequency in HI partners of DXN mating (%)	No of alleles in normal partners of DXN mating (n=86)	Frequency in normal hearing partners of DXN (%)	No. of alleles in normal hearing control (n=330)	Frequency in Normal hearing controls (%)
	CODON	PROTEIN									
1	c.71 G>A	p.W24X	TM1	60	25.42	28	30.43	6	6.98	6	1.82
2	c.79 G>A	p.V27I	TM1	2	0.85	0	0	0	0	0	0
3	c.104 T>G	p.I35S	TM1	2	0.85	0	0	0	0	0	0
4	c. 109 G>A	p.V37I	TM1	1	0.42	0	0	0	0	0	0
5	c. 126 G>T	p.E42D	EC1	3	1.27	0	0	0	0	0	0
6	c.135 A>G	p.G45G	EC1	0	0	0	0	0	0	1	0.3
7	c.165 C>A	p.T55T	EC1	1	0.42	0	0	0	0	3	0.91
8	c. 185 A>G	p.N62S	EC1	0	0	0	0	0	0	1	0.3
9	c. 224 G>A	p. R75Q	TM2	1	0.42	0	0	0	0	0	0
10	c.231 G>A	p.W77X	TM2	5	2.12	1	1.09	0	0	0	0
11	c.240 G>A	p.Q80Q	TM2	0	0	0	0	0	0	2	0.61
12	c.257C>T	p.T86M	TM2	0	0	2	2.17	1	1.16	0	0
13	c.262 G>A	p.A88A	TM2	0	0	2	2.17	1	1.16	0	0
14	c.341 A>G	p.E114G	IC2	2	0.85	0	0	0	0	0	0
15	c. 370 C>T	p.Q124X	IC2	3	1.27	2	2.17	1	1.16	0	0
16	c.380 G>A	p.R127H	IC2	25	10.59	4	4.35	6	6.98	57	17.27
17	c.439 G>A	p.E147K	TM3	0	0	0	0	0	0	1	0.3
18	c.457 G>A	p.V153I	TM3	14	5.93	1	1.09	3	3.49	6	1.82
19	c.493 C>T	p.R165W	EC2	1	0.42	0	0	1	1.16	3	0.91
20	c. 551G>A	p.R184Q	EC2	1	0.42	0	0	0	0	0	0
21	c.585 G>A	p.M195I	TM4	0	0	1	1.09	0	0	1	0.3
22	c.675 A>T	p.P225P	IC3	0	0	1	1.09	0	0	1	0.3
23	c.IVS 1+1 G>A (-3172 G>A)		Intronic Splice site region	4	1.7	0	0	0	0	0	0
TOTAL				125	52.96	42	45.65	19	22.09	82	24.85

TM1-4 – Transmembrane domain 1 -4; EC1 & EC2 – extracellular domains 1 & 2; IC – cytoplasmic domain

**Table 5:**
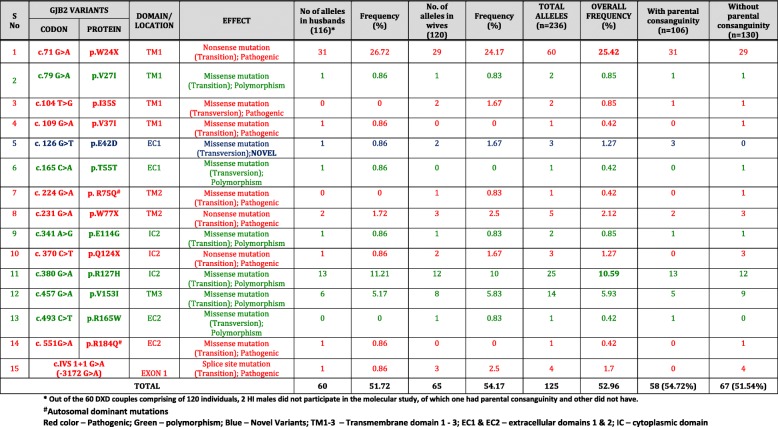
Summary of pathogenic mutations/ variants in the *GJB2* gene observed among the HI mates of 60 DXD families

Two dominant mutations, p.R75Q [11] and p.R184Q [12] were recorded for the first time in the Indian population through our study. The variants, p.V37I, p.T55 T and p.R165W were represented only once in this cohort.

Among the 46 DXN families, 46 HI individuals and 43 normal hearing mates were included in the molecular analysis. On *GJB2* mutation screening, 56.52% (26/46) of the HI partners and 41.86% (18/43) of the normal hearing partners had at least one nucleotide change in the *GJB2* gene. The *GJB2* mutations/variants observed among the affected and normal partners in DXN mating has been tabulated separately in Table 6. Ten different mutations/ variants in the *GJB2* gene were observed among the participating mates of 46 DXN families. A rare variant p.T86M was observed for the first time in the Indian population. The mutations/ variants, p.W77X, p.A88A, p.V153I, p.R165W, p.M195I and p.P225P were represented only once in this cohort. The overall allelic frequency of *GJB2* variants among the HI partners was 45.65%, which was lower than the frequency observed in DXD mates. Interestingly, the allelic frequency of *GJB2* variants in HI individuals with parental consanguinity (33.33%) was much lower than those without parental consanguinity (59.09%). The allelic frequency of *GJB2* variants in the normal hearing partners was 22.09%, is comparable with that observed in the normal hearing controls (24.85%). The allelic frequency is higher in normal hearing mates with parental consanguinity (30%) than those without parental consanguinity (21.05%), similar to that observed in DXD mates.

**Table 6:**
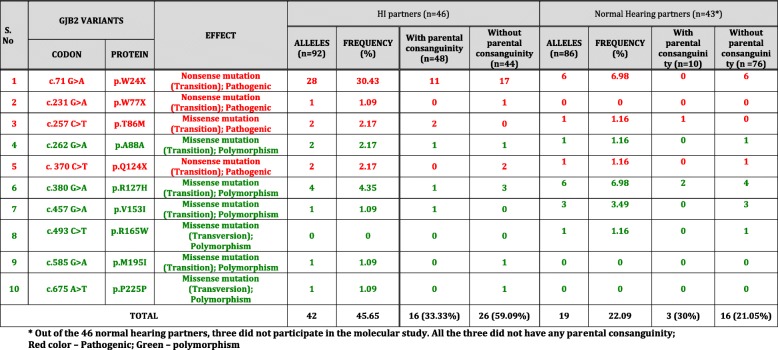
Summary of pathogenic mutations/ variants in the *GJB2* gene observed among the HI and Normal hearing partners of 46 DXN families

The frequency of pathogenic *GJB2* mutations was 33.90% in DXD mating (Table 5) and 35.86% in DXN mating (Table 6) with a combined frequency of 34.45%. Among them, p.W24X was the most common mutation at a frequency of 25.42% in DXD mates and 30.43% in the affected members of DXN mating with a combined frequency of 27.93%. More than 75% of the pathogenic alleles in this study had p.W24X mutation.

### Outcome of *GJB6* mutation screening

Hearing impaired individuals from both the types of mating, DXD and DXN, who were homozygous or compound heterozygous for pathogenic mutations in the coding and non-coding region of *GJB2* gene were excluded for further screening. Thus, 33 HI DXD mates and 14 affected partners of DXN mating were excluded. One hundred and seventeen HI individuals comprising of 85 DXD mates and 32 affected DXN mates were included for further screening for mutations in the *GJB6* gene. These included individuals with novel variants in the *GJB2* gene, heterozygous carriers of pathogenic mutations in the *GJB2* gene or negative for pathogenic *GJB2* mutations. In addition, 110 normal hearing controls were also included for the study of *GJB6* gene mutations.

#### Large deletions- 309 kb deletion (GJB6-D13S1830) & 232 kb deletion (GJB6-D13S1854) in *GJB6* gene

All the 117 HI individuals (85 DXD mates and 32 HI mates of DXN mating) and 110 normal hearing controls were negative for both the deletions, D13S1830 and D13S1854 in the *GJB6* gene, checked by multiplex PCR method [6, 7].

#### Point mutations in *GJB6* gene

Table 7 lists the *GJB6* variants observed in the 85 DXD mates and 32 HI partners of DXN mating by direct sequencing of the coding exon 6 of *GJB6* gene. Three novel variants, p.Q57R, p.E101Q and p.R104H were observed in heterozygous condition in 4 individuals. These three novel variants were found in heterozygous condition in three DXD mating individuals and one DXN affected member. These novel variants are reported for the first time ever in the HI population. Figure 1 shows the partial chromatograms of novel variants observed in *GJB6* gene.

**Table 7:** Novel *GJB6* variants observed in DXD and DXN families

S. No.	*GJB6* Variants		Domain/ Location	Effect	Alleles in DXD (n=170)*	Alleles in DXN (n=64)*	Overall Allelic Frequency (%)
	Codon	Protein					
1	c.311 G>A	p.R104H	IC2	Missense mutation; Transition; NOVEL; Possibly pathogenic	2 (1.18%)	0	0.85%
2	c.170 A>G	p.Q57R	EC1	Missense mutation; Transition; NOVEL; Possibly pathogenic	1 (0.59%)	0	0.43%
3	c.301 G>C	p.E101Q	IC2	Missense mutation; Transversion; NOVEL; Possibly pathogenic	0	1 (1.56%)	0.43%

* HI mates with novel variants in the *GJB2* gene, heterozygous carriers of pathogenic mutations in the *GJB2* gene or negative for pathogenic *GJB2* mutations were included for *GJB6* mutation screening

### Genotypes of *GJB2* & *GJB6* mutations

The various *GJB2* genotypes observed among the affected partners of 60 DXD families are listed in Table 8. Two mutations, p.W24X and p.W77X, a novel variant p.E42D, and polymorphisms, p.R127H and p.V153I, were found in homozygous state. Homozygous p.W24X was the most common pathogenic genotype observed with an overall frequency of 22.03%. Two HI individuals showed triallelic combinations, with one having a rare triallelic combination R184Q/Q124X/ IVS1 + 1G > A, involving a dominant mutation p.R184Q [12] and another having a combination of W24X/T55T/R127H. In one individual, the novel variant p.E42D was also found in combination with another novel variant, p.R104H in the second auditory gene analyzed, *GJB6*, showing digenic inheritance.

**Table 8:** Frequency and distribution of *GJB2* and *GJB6* genotypes observed among the 118 hearing impaired mates of DXD mating

S. No.	*GJB2* and *GJB6* genotypes	HI Husband (n=58)*	Frequency %	HI Wife (n=60)	Frequency %	Total (n=118)	Combined Frequency (%)
I	*GJB2*-Biallelic & Triallelic						
1	W24X/W24X	13	22.41	13	21.67	26	22.03
2	V153I/V153I	1	1.72	2	3.33	3	2.54
3	W77X/W77X	1	1.72	1	1.67	2	1.7
4	R127H/R127H	1	1.72	1	1.67	2	1.7
5	R127H/V153I	1	1.72	1	1.67	2	1.7
6	V27I/E114G	1	1.72	1	1.67	2	1.7
7	E42D/E42D	0	0	1	1.67	1	0.85
8	W77X/Q124X	0	0	1	1.67	1	0.85
9	W24X/I35S	0	0	1	1.67	1	0.85
10	Q124X/IVS1+1G>A	0	0	1	1.67	1	0.85
11	R75Q^#^/V153I	0	0	1	1.67	1	0.85
12	V37I/V153I	1	1.72	0	0	1	0.85
13	V153I/R165W	0	0	1	1.67	1	0.85
14	R184Q^#^/Q124X/IVS1+1G>A	1	1.72	0	0	1	0.85
15	W24X/T55T/R127H	1	1.72	0	0	1	0.85
II	*GJB2*-Monoallelic						
1	R127H/+	9	15.52	9	15	18	15.25
2	W24X/+	4	6.9	2	3.33	6	5.08
3	V153I/+	2	3.45	1	1.67	3	2.54
4	IVS1+1G>A/+	0		2	1.67	2	1.7
5	I35S/+	0		1	1.67	1	0.85
III	*GJB6*-Monoallelic						
1	R104H/+	1	1.72	0	0	1	0.85
2	Q57R/+	1	1.72	0	0	1	0.85
IV	*GJB2/GJB6*-Digenic						
1	E42D/+; R104H/+	1	1.72	0	0	1	0.85

* Out of the 60 DXD couples comprising of 120 individuals, 2 individuals did not participate in the molecular study

^#^Autosomal dominant mutations

The various *GJB2 & GJB6* genotypes observed among the affected partners and the normal hearing partners of 46 DXN families have been listed in Table 9. Three mutations, p.W24X, p.Q124X and p.T86M, have been found in homozygous state. Homozygous p.W24X is the most common pathogenic genotype observed in this sub group also with a frequency of 23.91%, which is marginally higher than in DXD mating. The *GJB2* genotypes observed among the control group have been listed out in Table 10 No *GJB6* variants were observed among the normal hearing controls.

**Table 9:** Frequency and distribution of *GJB2* and *GJB6* genotypes observed among the mates of DXN mating

S. No.	Genotypes	Affected Partner (n= 46)	Frequency (%)	Normal hearing partner (n=43)*	Frequency (%)
I	*GJB2*-Biallelic				
1	W24X/W24X	11	23.91	0	0
2	Q124X/Q124X	1	2.17	0	0
3	T86M/T86M	1	2.17	0	0
4	W24X/W77X	1	2.17	0	0
5	W24X/A88A	1	2.17	0	0
6	R153I/R165W	0		1	2.33
6	M195I/P225P	1	2.17	0	0
II	*GJB2*-Monoallelic				
1	W24X/+	4	8.7	6	13.95
	T86M/+	0	0	1	2.33
	Q124X/+	0	0	1	2.33
2	R127H/+	4	8.7	6	13.95
3	V153I/+	1	2.17	2	4.65
4	A88A/+	1	2.17	1	2.33
III	*GJB6*-Monoallelic				
1	E101Q/+	1	2.17	0	0

* Out of the 46 normal hearing partners, three did not participate in the molecular study. All the three did not have any parental consanguinity

**Table 10:** Frequency and distribution of *GJB2* genotypes observed among the 165 normal hearing controls

S.No.	*GJB2* genotype	Normal hearing control population (n=165)	Frequency %
1	W24X/+	5	3.02
2	N62S/+	1	0.61
3	E147K/T55T	1	0.61
4	W24X/M195I/P225P	1	0.61
1	R127H/R127H	5	3.02
2	R127H/R165W	1	0.61
3	R127H/T55T	1	0.61
4	V153I/R165W	2	1.21
5	Q80Q/R127H	1	0.61
6	R127H/+	44	26.67
7	V153I/+	4	2.41
8	Q80Q/+	1	0.61
9	T55T/+	1	0.61
10	G45G/+	1	0.61
		69	41.82

The overall carrier frequency for *GJB2* pathogenic mutations, including the novel variants, among the HI mates, in both the sub groups included (DXD and affected partners of DXN mating), was 4.88%, which was twice the frequency observed in the normal hearing controls (2.42%). The carrier frequency of normal hearing partners of DXN mating was as high as 9.30%.

### Novel variants in *GJB2* and *GJB6* genes

**Fig. 2:**
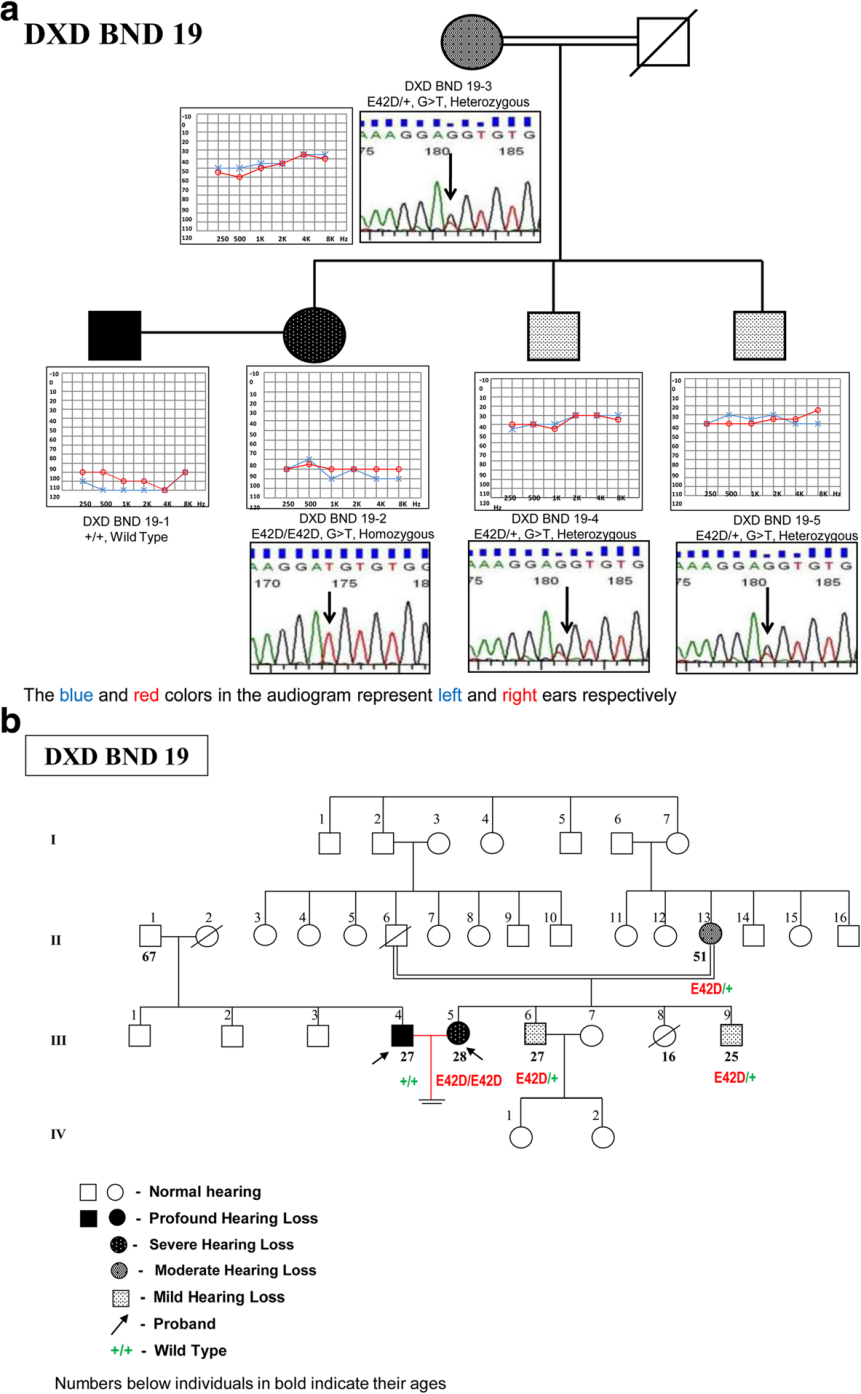
**a** Genotype-Phenotype correlation in DXD BND 19 family with novel *GJB2* mutation. **b** Pedigree of DXD BND 19 family showing novel mutation p.E42D in *GJB2* gene

Four novel variants, p.E42D (in *GJB2* gene), p.Q57R, p.E101Q, p.R104H (in *GJB6* gene) were identified in this study. This is the first study from Indian subcontinent reporting novel variants in the coding region of *GJB6* gene.

p.E42D was observed at a frequency of 1.27% among the DXD mates. It is a missense mutation due to G > T transversion at 126th nucleotide, resulting in a change from glutamic acid to aspartic acid at 42nd codon in the EC1 domain of the protein. It was observed in two DXD families, in homozygous, heterozygous as well as in compound heterozygous state along with a novel *GJB6* mutation and variable phenotypes. In the first DXD family (DXD BND19, Fig. 2a) it was observed in homozygous state (E42D/E42D) in the female DXD mate & in heterozygous state among her two siblings and her mother, all of whom showed variable phenotypes ranging from mild to profound, conductive, sensorineural and mixed type of hearing losses (Fig. 2b). The affected members did not have any other associated clinical features. The members with mild and moderate hearing losses were not aware of their hearing status until our audiometric evaluation.

**Fig. 3:**
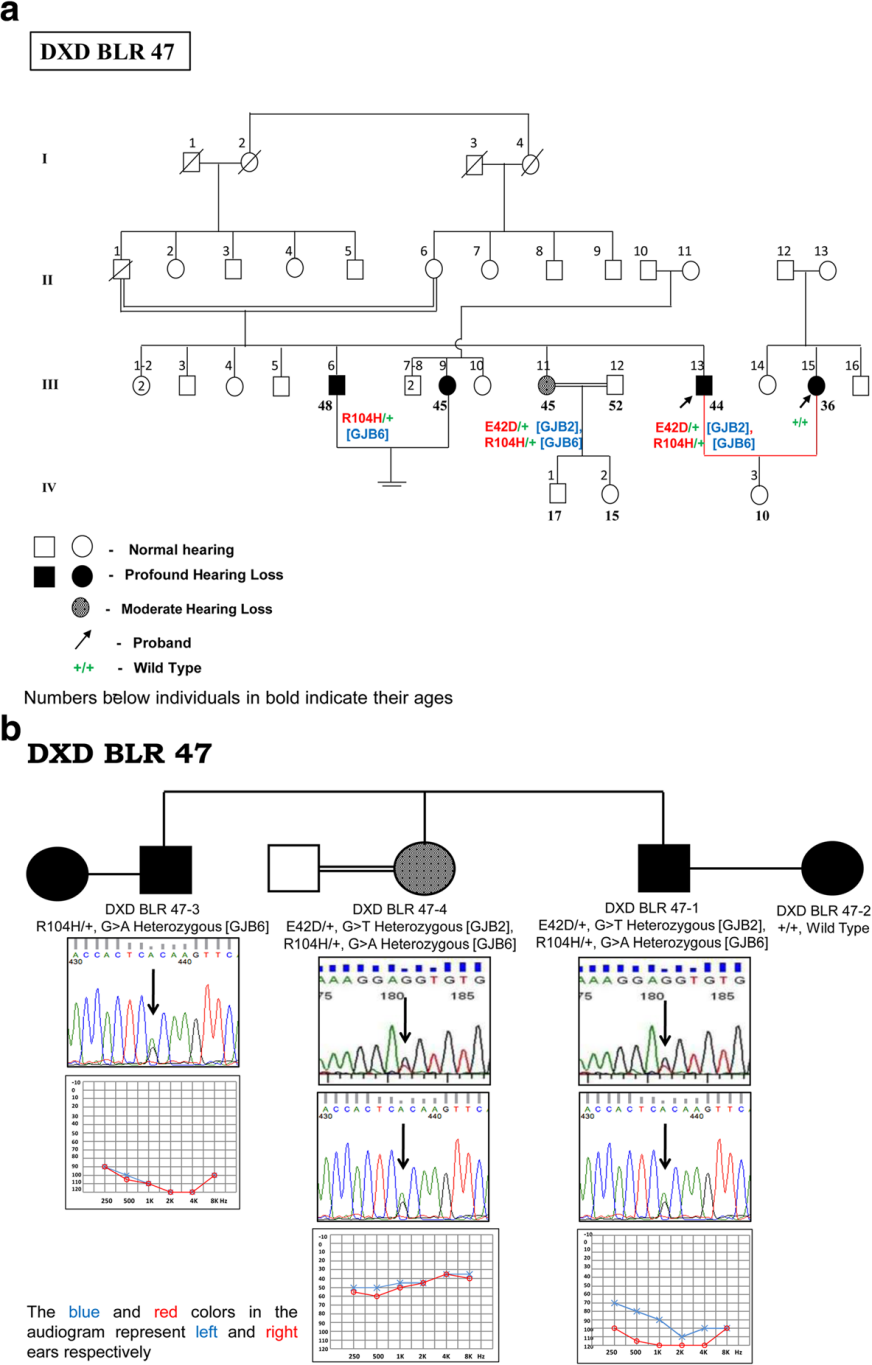
**a** Pedigree of DXD BLR 47 family showing novel point mutations p.E42D& p.R104H in *GJB2*&*GJB6* genes, respectively (digenic). **b** Genotype-Phenotype correlation in DXD BLR 47 family with novel *GJB2/GJB6* mutations

In the second DXD family (DXD BLR47, Fig. 3a), the novel mutation was observed in compound heterozygous state (E42D/+) along with a novel *GJB6* gene mutation (R104H/+) in the HI husband, suggesting a digenic interaction between the two. The affected elder sister showed a similar genotype involving the two novel variants in the *GJB2* and *GJB6* genes. The affected elder brother did not have any changes in the *GJB2* gene and had only the novel variant in heterozygous condition (R104H/+) in the *GJB6* gene. Audiological evaluation of the affected husband, affected wife and affected elder brother showed them to have bilateral, profound, sensorineural hearing loss, while the affected elder sister had bilateral, moderately severe, sensorineural hearing loss (Fig. 3b). The affected elder brother had goiter, which appeared in the second decade of his life. The affected members did not have any other associated clinical features. p.R104H is a G > A transition at 311th nucleotide resulting in a change from arginine to histidine at the 104th codon in the IC2 domain of the connexin 30 protein (Fig. 1).

The novel missense variant p.R104H in the *GJB6* gene was observed at a frequency of 1.71% in the 117 HI individuals selected for the second level screening, in this study.

#### p.Q57R variant (in *GJB6* gene)

**Fig. 4:**
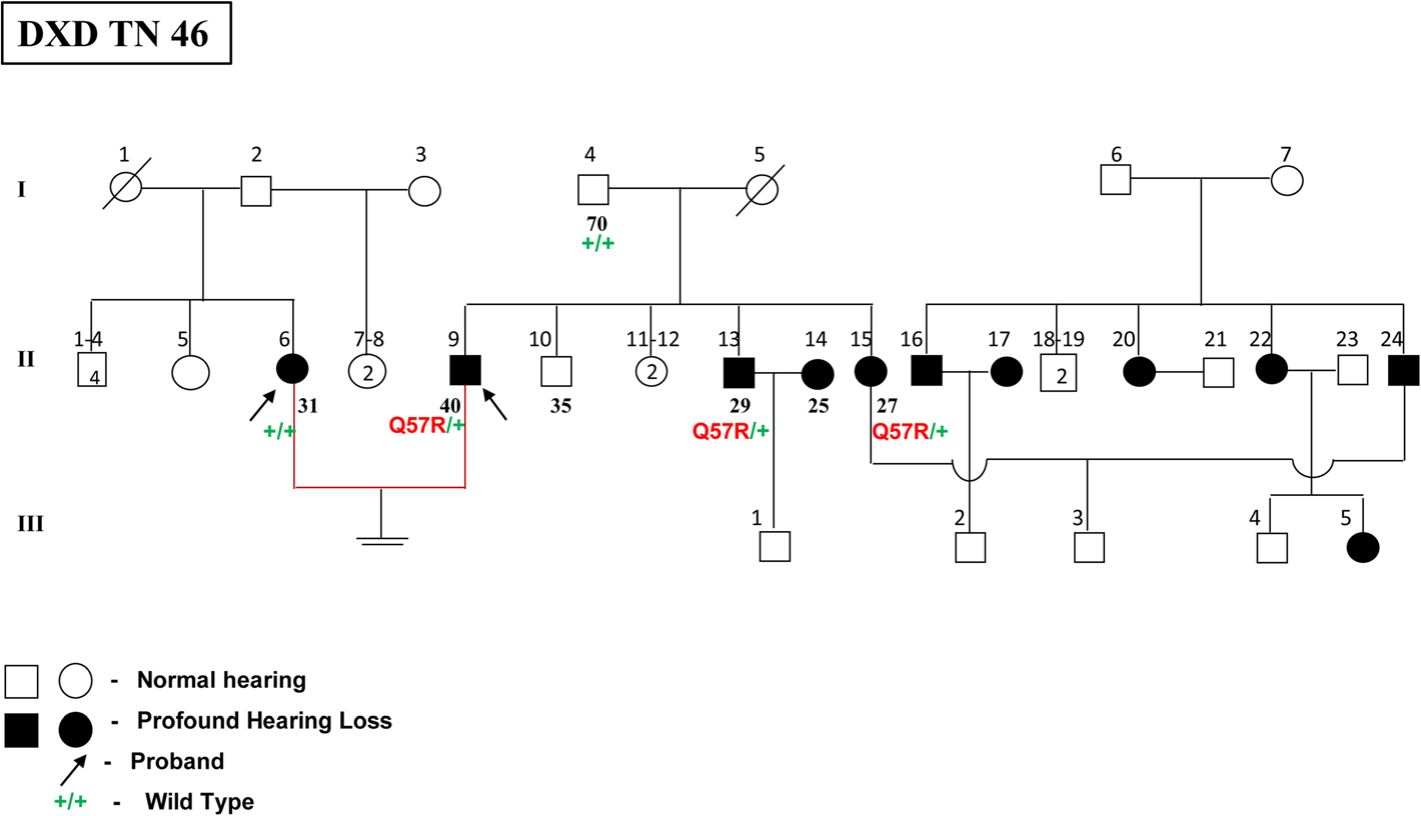
Pedigree of DXD TN 46 family showing novel mutation p.Q57R in *GJB6* gene

p.Q57R, a **novel missense variant**, was observed in the *GJB6* gene at a frequency of 0.85% in the 117 HI individuals. This variant was observed in only one DXD mate in a heterozygous condition (Q57R/+) with no associated *GJB2* gene mutations (Fig. 4). The DXD couple did not have any changes in the *GJB2* gene. The affected husband, his affected brother and sister had a novel variant p.Q57R in heterozygous condition (Q57R/+) in the *GJB6* gene. The affected wife did not have any changes in the *GJB6* gene. This novel mutation was not observed in the selected DXN mates and the normal hearing controls. p.Q57R is an A > G transition at 170th nucleotide resulting in a change from glutamine to arginine at the 57th codon in the EC1 domain of the connexin 30 protein (Fig. 1).

#### p.E101Q variant (in *GJB6*gene)

**Fig. 5:**
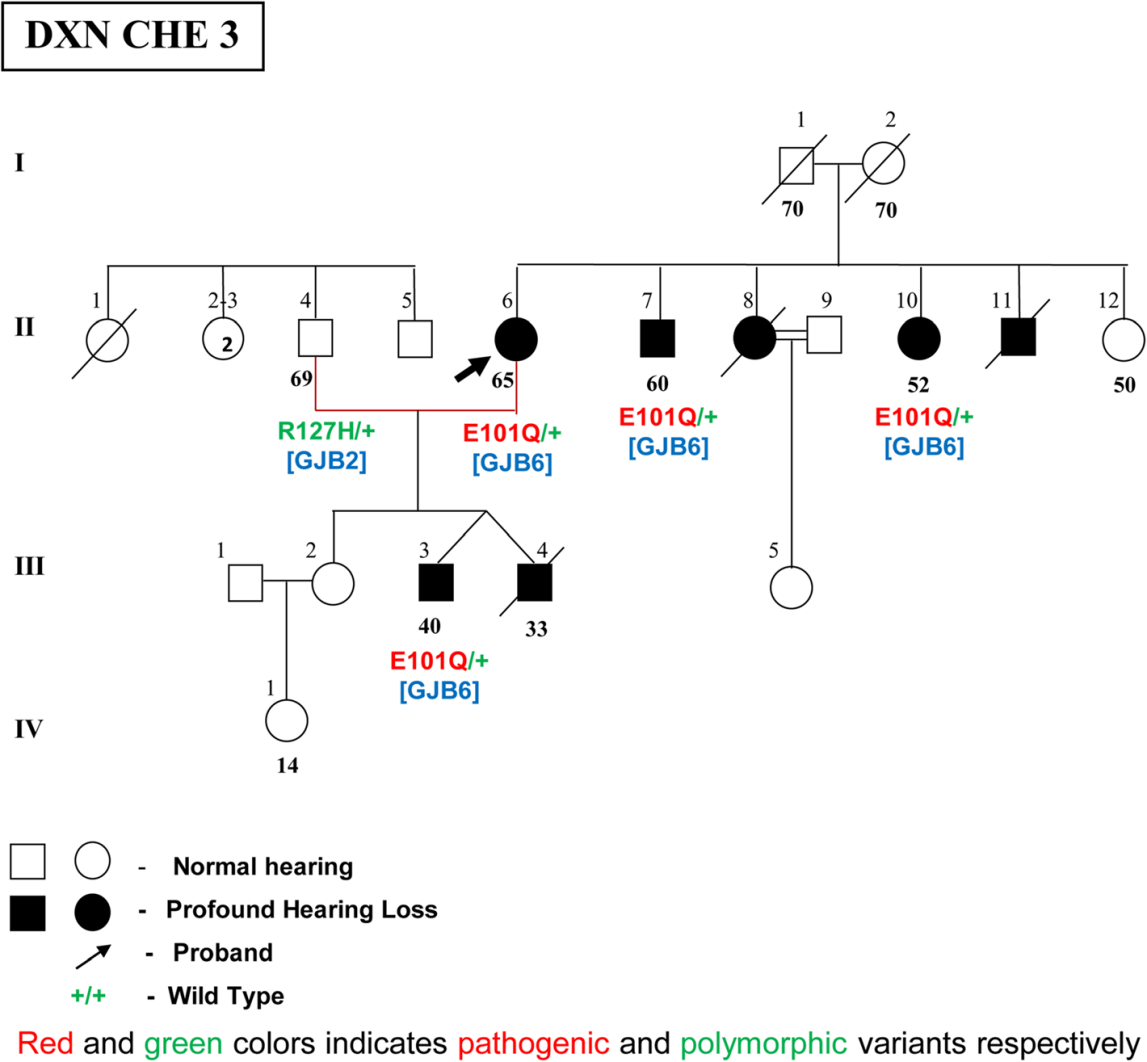
Pedigree of DXNCHE3 family showing novel mutation p.E101Q in *GJB6* gene

p.E101Q is a G > C transition at the 301st nucleotide resulting in a change from glutamic acid to glutamine at the 101st codon in the IC2 cytoplasmic domain of the connexin 30 protein (Fig. 1). This novel missense variant in the *GJB6* gene was observed at a frequency of 0.85% in 117 HI individuals. It was observed in only one affected female partner of a DXN family in a heterozygous condition (E101Q/+) with no associated *GJB2* mutations (Fig. 5).There was no parental consanguinity in both the sides, but the wife’s side had history of deafness with four siblings affected. The couple had two affected monozygotic twins, but one of them died due to unspecified illness. The couple also had a normal hearing daughter, who did not participate in the study. The DXN couple, two affected siblings of the proband and the surviving affected son participated in the study. Audiological evaluation of the four affected members showed them to have bilateral, profound, sensorineural hearing loss. The affected members did not have any other associated clinical features. The HI wife, her HI son and her two HI siblings did not have any changes in the *GJB2* gene. They all had the novel variant p.E101Q in the *GJB6* gene in heterozygous condition (E101Q/+).It was absent in the 85 DXD mating individuals and the normal hearing controls. The normal hearing husband of the deaf mate in this DXN family was a carrier of the common polymorphism p.R127H in the *GJB2* gene in heterozygous condition (R127H/+), which was also present in the affected son.

### In silico analysis of novel variants observed in *GJB2* and *GJB6* genes

We predicted the functional significance of the novel variant p.E42D identified in *GJB2* and the 3 novel variants, p.Q57R, p.E101Q and p.R104H, identified in *GJB6* using two in silico tools namely SIFT and PolyPhen2. Predictions by the former tool is based on the alignment of orthologous and/or paralogous protein sequences while the latter considers evolutionary conservation, the physiochemical differences, and the proximity of the substitution to predicted functional domains and/or structural features. The outputs of both tools show that p.Q57R and p.R104H may affect or damage the structure and functioning of the Cx30 protein, but p.E101Q is tolerable or benign. Also, p.E42D in *GJB2* was predicted to be tolerable or benign (Table 11).

**Table 11:** Comparative analysis of the SIFT predictions for the novel variants in *GJB2* and *GJB6* genes

Mutation	SIFT				PolyPhen-2					
					HumDiv			HumVar		
	Score	Median Sequence conservation	Sequences represented at position	Comment	Score	Sensitivity, Specificity	Comment	Score	Sensitivity, Specificity	Comment
p.E42D (*GJB2*)	0.57	3.05	42	Tolerated	0.038	0.94, 0.82	Benign	0.052	0.93, 0.63	Benign
p.Q57R (*GJB6*)	0.00	3.09	35	Affect protein function	1.000	0.00, 1.00	Probably damaging	1.000	0.00, 1.00	Probably damaging
p.E101Q (*GJB6*)	0.48	3.10	29	Tolerated	0.183	0.92, 0.87	Benign	0.114	0.90, 0.69	Benign
p.R104H (*GJB6*)	0.01	3.09	35	Affect protein function	0.990	0.41, 0.98	Probably damaging	0.749	0.77, 0.86	Possibly damaging

To further gain insight on the effect of these mutations on the physicochemical parameters of Cx26 or Cx30 protein, we analysed their native and mutant structures individually in Expasy’s ProtParam tool and compared the results (Table 12).

**Table 12:** Comparison of native and the mutant structure with p.E42D variant in Cx26 protein using Expasy’s ProtParam tool

	*GJB2*		*GJB6*			
Property	Native	p.E42D	Native	p.Q57R	p.E101Q	p.R104H
Number of amino acids	226	226	261	261	261	261
Molecular weight	26215	26201	30387.4	30415.5	30386.4	30368.4
Theoretical pI (Isoelectric point)	9.11	9.11	8.81	8.92	8.92	8.68
Total number of negatively charged residues (Asp + Glu)	18	18	23	23	22	23
Total number of positively charged residues (Arg + Lys)	27	27	29	30	29	28
Total number of atoms	3721	3718	4274	4280	4275	4268
Ext. coefficient assuming all pairs of Cys residues form cystines	52410	52410	52410	52410	52410	52410
Ext. coefficient assuming all Cys residues are reduced	51910	51910	51910	51910	51910	51910
Estimated half life (mammalian reticulocytes, in vitro)	30 hrs	30 hrs	30 hrs	30 hrs	30 hrs	30 hrs
Instability index	42.8	42.8	43.11	42.60	43.11	44.01
Aliphatic index	98.67	98.67	91.07	91.07	91.07	91.07
Grand average of hydropathicity (GRAVY)	0.288	0.288	0.055	0.051	0.055	0.06

The native and the mutant proteins differ in their molecular weight and the number of atoms they are composed of. Moreover, the total number of positively charged residues increases from 29 to 30 in case of p.Q57R. On the other hand, p.R104H decreases the positively charged residues from 29 to 28. The mutation p.E101Q reduces the number of negatively charged residues from 23 to 22. The instability index of p.Q57R and p.R104H, shows a slight variation when compared to that of the native protein. p.Q57R tends to slightly increase the stability of the protein and in contrast p.R104H makes the protein more unstable. However, an instability value > 40 suggests that the mutant forms as well as the native form are quite unstable since they are membrane channels. Moreover, a shift was observed in the GRAVY score for the mutants p.Q57R and p.R104H. Positive scores of GRAVY indicate the hydrophobic nature of the protein. p.Q57R reduces the hydrophobicity while p.R104H increases this property. There is no change in the aliphatic index and this high score (91.07) suggests that the native and the mutant proteins may retain their conformation over a wider range of temperatures.

**Fig. 6:**
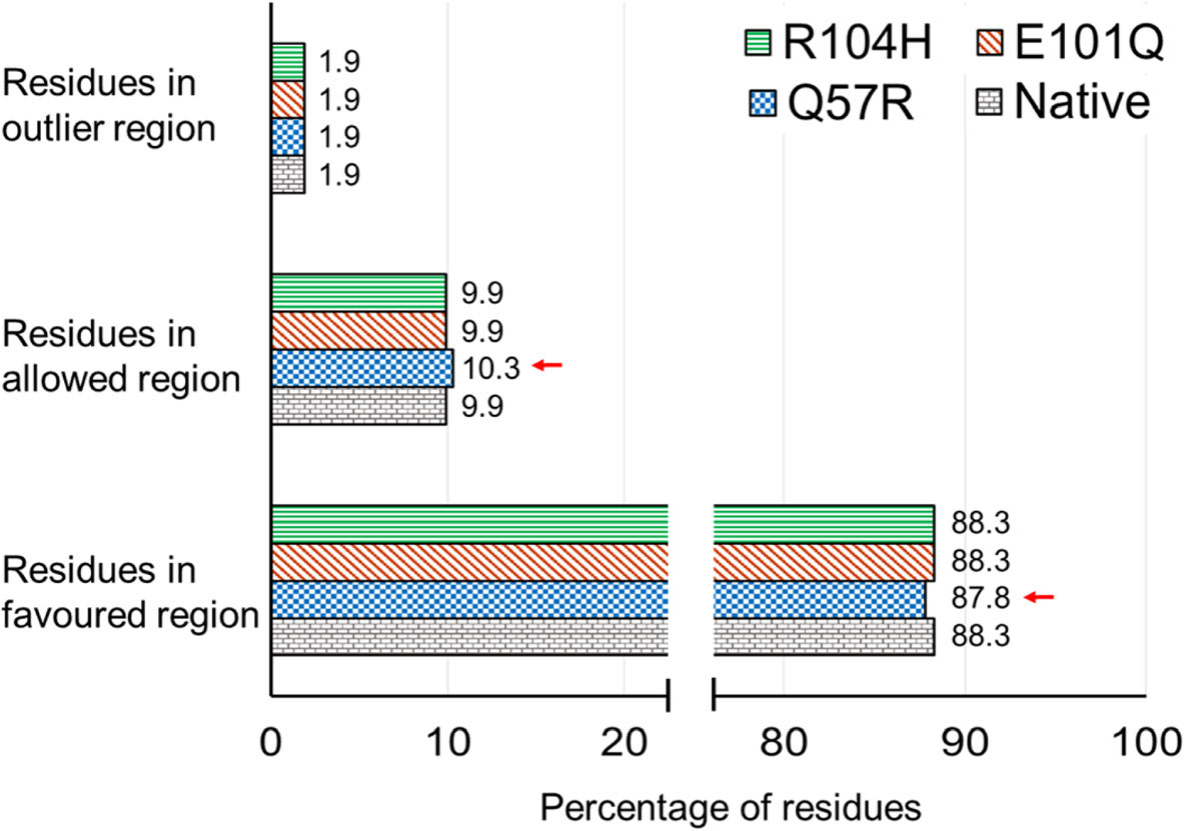
Residue profiling of native and mutated connexin 30 calculated by RAMPAGE

The function of a protein not only relies on its properties but also on its three dimensional structure. Hence we queried RCSB Protein Data Bank for the 3-dimensional structures of Cx26 and Cx30 proteins. Only the crystal structure of Cx26 was available, which was downloaded and used for analyzing the effect of p.E42D mutation. The model was viewed with the help of Swiss-PdbViewer. The Glutamic acid at position 42 was found to be a part of an alpha helix and it was mutated to Aspartic acid in all 6 chains of the hexameric protein. Both the mutated and the native structures were subjected to Ramachandran Plot analysis through RAMPAGE Online portal. The results showed that the mutation p.E42D did not alter the percentage of amino acid residues in the favorable, allowed and outlier regions of the plot (Fig. 6).

**Fig. 7:**
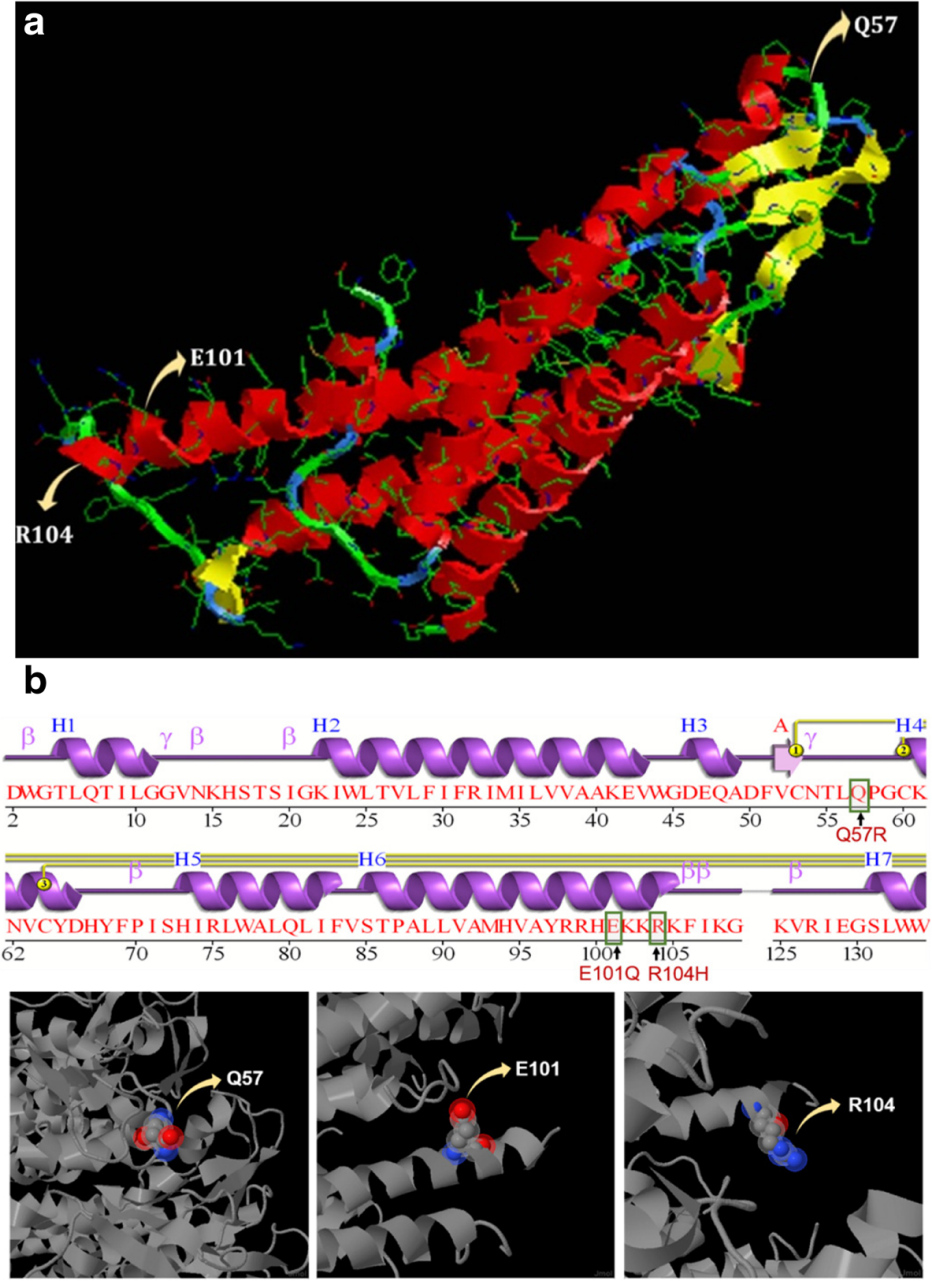
**a** Predicted model of connexin 30 protein single chain indicating the positions of the variants, Q57, E101 & R104 observed in this study. **b**: Wiring diagram and 3-D structure of connexin 30 protein showing the position of Q57, E101 and R104

Connexin 30 structure was also modeled using SWISS MODEL repository. As expected, the QMEAN Z-score of the model was low (− 6.59), reinforcing the fact that it is a membrane protein. The predicted structure of Cx30 protein is shown in Fig. 7a and b with 7 Helices, 8 Strands and 13 Turns. In this model, Glutamic Acid (E) at position 101 and Arginine (R) at position 104 are part of alpha helices, while Glutamine (Q) at position 57 was found in the loop region that connects a beta sheet with alpha helix.

The mutant structures of Cx30 with the substituting amino acid at the above mentioned positions (p.Q57R, p.E101Q and p.R104H) were created with the help of Swiss-PdbViewer and saved as separate files. These structures were energy minimized and submitted to RAMPAGE online portal. Computation of Ramachandran plot showed that only p.Q57R is capable of changing the conformation of the protein, since the substitution decreased the number of residues in the favored region and increased the number of residues in the allowed region (Fig. 6).

### Genotype-phenotype correlation of *GJB2*/*GJB6* mutations in the incidence of hearing loss in DXD families

The 60 DXD families are further classified based on the genotype-phenotype correlation in the offspring with respect to the *GJB2* and *GJB6* mutations, as listed in Table 13. The table divides the 60 DXD mating families further into four groups: Group-I: Non-complementary mating families with all affected offspring only Group-II: Complementary mating families with all hearing offspring only Group-III: Segregating type families with both affected and normal hearing offspring Group-IV: Families with no offspring.

**Table 13:** Genotype-phenotype correlation of *GJB2/GJB6* mutations in the offspring of DXD mating

		BOTH PARTNERS HAVING *GJB2*			ONLY ONE PARTNER WITH *GJB2*		ONLY ONE PARTNER WITH *GJB6*	ONLY ONE PARTNER WITH *GJB2/GJB6*		
S. No.	SUBGROUPS IN DXD MATING BASED ON PHENOTYPE OF OFFSPRING	BOTH HOMOZYGOUS OR COMPOUND HETEROZYGOUS	BOTH HETEROZYGOUS	ONE HOMOZYGOUS, ONE HETEROZYGOUS	HOMOZYGOUS	HETEROZYGOUS	ONE HETEROZYGOUS	DIGENIC	NON-*GJB2* & NON-*GJB6*	TOTAL (%)
1	Non-complementary with all deaf offspring	8	0	0	1	1	1	0	6*	17 (28.33%)
2	Complementary with all hearing offspring	0	1	1	11	6	0	1	10^#^	30 (50%)
3	Mixed offspring (hearing and hearing impaired)	0	0	0	0	1	0	0	1	2 (3.33%)
4	NO OFFSPRING	0	0	1	4	0	1	0	5	11 (18.33%)
5	TOTAL	8	1	2	16	8	2	1	22	60

*One HI male partner did not participate in the study, but his offspring did not carry any *GJB2* mutations

^#^One HI male partner expired, but his offspring did not carry any *GJB2* mutations

#### Group I

Seventeen families (28.33%) belonged to this group where all offspring were affected. In 8 out of these 17 families, both the affected partners had *GJB2* mutations in either homozygous condition or in compound heterozygous condition. In other words, 47.06% of the non-complementary matings were caused by *GJB2* mutations. Overall, 13.33% of the DXD families were affected by *GJB2* mutations. One among these 8 families had a unique triallelic pattern involving both a dominant mutation as well as recessive mutations of *GJB2* gene (R184Q/ Q124X/ IVS1 + 1G > A), observed for the first time in the world [12].

In 3 other families, only one of the two deaf partners had either a homozygous, or heterozygous *GJB2* mutation or a heterozygous *GJB6* mutation, but had HI offspring. In the remaining 6 families, none of the partners had any *GJB2*/*GJB6* mutations.

#### Group II

Thirty families (50%) in this group had all normal hearing offspring. Eleven out of these families did not have any *GJB2* or *GJB6* mutations. In the remaining 19 families (63.33%), one family had a combination of one deaf partner having *GJB2* mutation in homozygous condition and other partner having a *GJB2* mutation in heterozygous condition, but having normal hearing offspring. In the remaining 18 families, only one partner had a single *GJB2* mutation in homozygous condition or heterozygous condition, or a *GJB6* mutation in heterozygous condition.

#### Group III

Two families (3.33%) out of the 60 DXD families had one normal hearing and one affected offspring each. One family was a non-*GJB2* family with both the HI partners not having any *GJB2* mutations. In the second family in this group, one of the deaf partners had an autosomal dominant *GJB2* mutation in heterozygous condition (R75Q/+) with the affected offspring also inheriting the same from the parent [11].

#### Group IV

Eleven families (18.33%) out of the 60 DXD families did not have any offspring. Five out of these families did not have any *GJB2* or *GJB6* mutations. In 1 out of the remaining 6 families, one deaf partner had homozygous *GJB2* mutation while the other had in heterozygous condition. In the remaining 5 families, 4 families had one partner with a homozygous *GJB2* mutation and the remaining one had one partner with novel *GJB6* mutation in heterozygous condition (Q57R/+).

### Role of *GJB2*/*GJB6* mutations in the incidence of hearing loss in DXN families

The 46 DXN families were further classified based on consanguinity and the role of *GJB2*/ *GJB6* mutations in the incidence of hearing loss, as the principle of complementarity cannot be applied to this subgroup at the phenotypic level.

#### Group-I families: Consanguineously mating DXN families with and without *GJB2*/*GJB6* mutation affliction

This group consisted of 18 DXN families (39.13%), with consanguineous marriages. Twelve families (66.67%) had affected offspring, indicating the role of consanguinity in the incidence of hearing loss. Eight families (44.44%) had *GJB2*/*GJB6* mutations in one or both the partners in homozygous or heterozygous conditions (Table 14). Five out of these 8 families had affected offspring, two had normal hearing offspring and in one family there was no offspring.

**Table 14:**
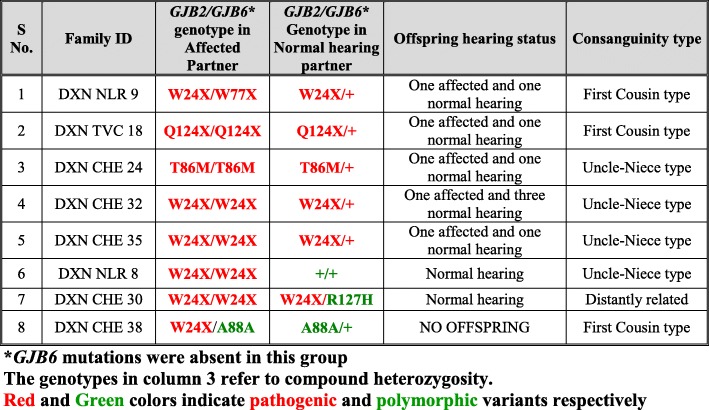
Consanguineously mating DXN Families (Group I) with *GJB2/GJB6* mutations

#### Group II families: Non-consanguineously mating DXN families with and without *GJB2*/*GJB6* mutation affliction

This group consisted of 28 DXN families (61.87%), with non-consanguineous mating. Seven out of the 28 families (25%) had affected offspring while 71.43% of these families had only normal hearing offspring (20/28). One family did not have offspring. Twelve out of the 28 families (42.86%) had *GJB2*/*GJB6* mutations in one or both the partners in homozygous or heterozygous conditions (Table 15). Out of these 12 families, two families (16.67%) had affected offspring, one of whom had a novel variant p.E101Q in the *GJB6* gene in heterozygous condition, reported for the first time through this study. In 7 families, the affected partners have the most common mutation p.W24X in homozygous condition (W24X/W24X), five of whom have normal hearing offspring and one does not have any offspring.

**Table 15:**
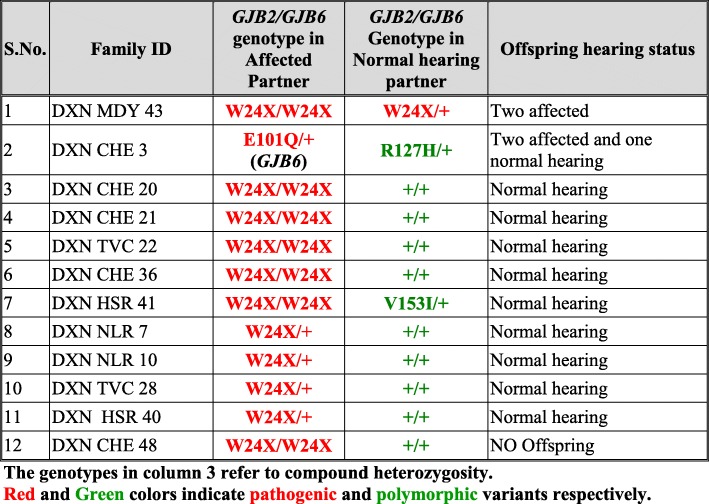
Non-consanguineously mating DXN families (Group II) with *GJB2/GJB6* mutations

### Statistical analysis for significance

The *GJB2* mutation frequency observed among the four study groups, (DXD mating, affected partners in DXN mating, normal hearing partners in DXN mating and the normal hearing controls) was compared using chi-square test, with the assumption that the differences if observed may be only due to chance. We observed that the chi-square value, which compares the differences between the observed and the expected values across the three groups, to be 58.21, with a *p*- value < 0.001, which is highly significant (Table 16).

**Table 16:** Chi-square analysis of *GJB2* variants among the four groups in our study

S. No.	Group		*GJB2* Positive Alleles	*GJB2* Negative Alleles	Total Alleles	Percentage (%)
1	DXD-Both Affected Partners	Observed (O)	125	111	236	52.96
		Expected (E)	85.01	150.99		
2	DXN- Affected Partners	Observed (O)	42	50	92	45.65
		Expected (E)	33.14	58.86		
3	DXN-Normal Hearing Partners	Observed (O)	19	67	86	22.09
		Expected (E)	30.98	55.02		
4	Control	Observed (O)	82	248	330	24.85
		Expected (E)	124.88	205.12		
	TOTAL		268	476	744	
	Chi-Square Value ( Σ(O-E)^2^/E)		58.21 (P< 0.001)			

## Discussion

Assortative mating as a form of non-random mating has the potential to act as an evolutionary agent. Assortative mating is capable of bringing together rare, non-allelic genes for the same phenotype, creating a non-random distribution of genes that has been termed “gametic-phase disequilibrium.” It also increases the population variance. However, as the number of genes involved in creating a particular trait increases, assortative mating has a reduced capability to increase homozygosity at any one locus. There have been several studies on assortative mating among the deaf in the US. Edward Allen Fay [28], through his monumental work “Marriages among the Deaf in America”, observed that marriages of the deaf had rapidly increased in America in that century, attributing largely to the establishment of schools for the deaf. Deaf marrying deaf constituted 72.5% of the married deaf population. Analysis of the DXD matings showed that 79% were “complementary” matings (i.e., only hearing offspring), 4.2% were “non-complementary” matings (capable of producing only deaf offspring), and the remaining 16.8% were “segregating” matings, in which the parents were capable of producing both deaf and hearing offspring.

This work was followed by another landmark work by Rose [29, 30] in which the data generated by Fay was compared with the contemporary data generated through a 1969 survey. Rose’s results showed that between the 19th and 20th centuries, the frequency of deaf children with one or two deaf parents increased by 38%. Among the deaf children through DXD mating, the estimated proportion of non-complementary marriages also increased by 23%. Based on these observations and using computer simulations, Nance et al. proposed that the introduction of sign language 400 years ago in many Western countries and subsequent establishment of residential schools for the deaf could have favored assortative mating among deaf and relaxed genetic selection against deafness, leading to doubling of frequency of DFNB1 deafness in the United States in the last 200 years [13, 14]. In a study on living deaf alumni of Gallaudet University, Arnos et al. [15] collected pedigree data on 311 marriages among deaf individuals. On the basis of segregation analysis on these 311 matings between deaf individuals, the authors reported that 23% were non-complementary, an increase of more than fivefold over the previous century’s data of 4.2%, as reported by Fay. Mutational analysis within these non-complementary mating individuals showed a statistically significant linear increase in the prevalence of pathologic *GJB2* mutations. In addition to this, 199 probands with one or both parents deaf were also screened for *GJB2* mutations and they too showed similar significant linear increase. In both these studies, c.35delG was the most common mutation in the *GJB2* gene, ranging from 69 to 73%. These data were consistent with the increase in the frequency of DFNB1 predicted by the previous simulation studies and provided convincing evidence over the influence of assortative mating on the frequency of common genes for deafness.

### DFNB1 dynamics in DXD families

In our present study, we observed 17 families out of the 60 DXD families (28.33%) to be non-complementary, i.e. with *all affected offspring*. This is higher than the Arnos et al.’s observation of 23%. While Arnos’ sampling was restricted to Gallaudet University alumni and not to a particular ethnicity or population, our study represents the south Indian HI population belonging to the four southern states-Andhra Pradesh, Karnataka, Kerala and Tamil Nadu. In 8 families out of the 17 non-complementary mating families, both the affected partners had *GJB2* mutations in either homozygous condition or in compound heterozygous condition (Table 8). Biallelic *GJB2* mutations accounted for 13.33% of the DXD families and 47.06% of the non-complementary families.

**Fig. 8:**
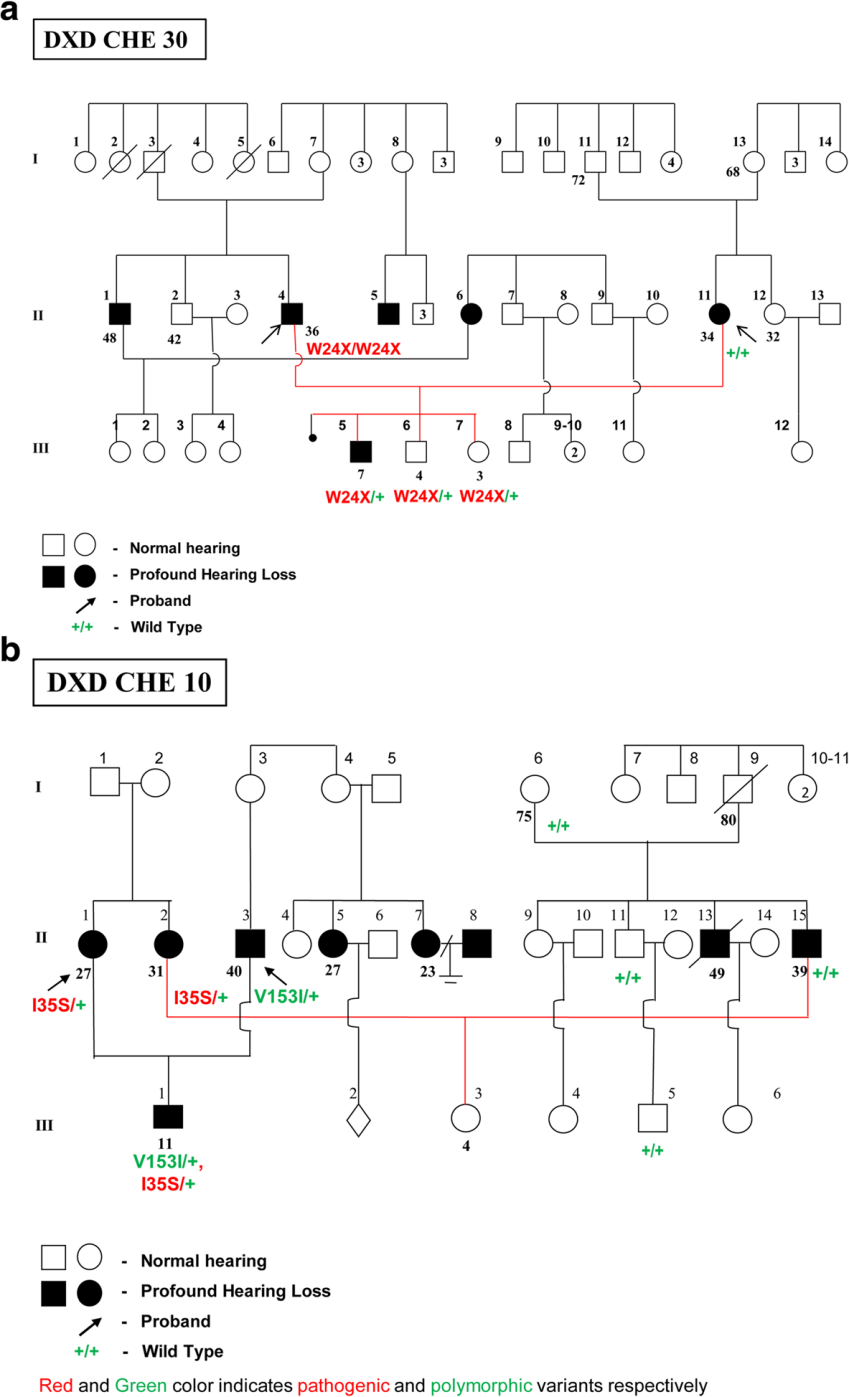
**a** Pedigree of DXD CHE 30 family wherein there is possibility of nonallelic gene interaction leading to hearing impairment. **b** Pedigree of DXD CHE 10 family wherein there is possibility of non-allelic gene interaction leading to hearing impairment

In three other families from our 17, only one of the two deaf partners had either a homozygous, or heterozygous *GJB2* mutation or a heterozygous *GJB6* mutation, but with HI offspring. In two of these families, the affected offspring had one copy of the pathogenic DFNB1 allele indicating the possibility of probable role of these mutations in the incidence of hearing loss. This phenomenon has been explained by Arnos et al. [15] through their observation on *GJB2* mutations among DXD families and their pedigree analysis. In their study, the pedigree analysis of such families suggested that many of the additional segregating matings reflect pseudo-dominant transmission in families when one parent with deafness resulting from *GJB2* and/or *GJB6* mutations married a partner who is deaf for reasons yet to be determined but was also a heterozygous carrier of a single *GJB2* or *GJB6* mutation. One of the important effect of assortative mating is the bringing together of rare, non-allelic genes (on different gene loci) for the same phenotype, creating a non-random distribution of genes that has been termed “gametic-phase disequilibrium.” In our study the possibility of co-occurrence of non-allelic genes in the affected offspring in at least two families (Fig. 8a and b) may be due to gametic phase disequilibrium.

One of the most important observations from this study is out of these eight biallelic *GJB2* mutation carrying non-complementary mating DXD families, 50% of them have p.W24X mutation in homozygous condition in both the partners. While one of these eight DXD pairs was related as first cousins, the remaining seven pairs were unrelated. This high prevalence of p.W24X mutations among the south Indian DXD families could be attributed to the high carrier rate (1.82%) and high parental consanguinity (45%) as observed in our study.

In our study, 50% of the 60 DXD families had *only normal hearing offspring* (complementary mating DXD families). In 19 of these DXD families, one of the mates had a *GJB2/GJB6* mutation in homozygous or heterozygous condition. p.W24X mutation was the most common mutation, with 11 out of these 19 mates having it in homozygous condition and 7 of them having in heterozygous condition. In these 11 matings, the non-*GJB2* partner possibly had mutations in genes that did not contribute towards an affected phenotype in the offspring in combination with the *GJB2* mutation. The remaining 7 mates with heterozygous condition could simply be carriers of p.W24X mutation but with cause for deafness lying in mutation or mutations in genes not associated with the DFNB1 locus. In the remaining one member, two novel *GJB2* and *GJB6* variants were observed in heterozygous condition showing digenic interaction, but his partner could be having a gene with no interaction with DFNB1 deafness causative factors.

In our study, 3.33% of 60 DXD families were of *segregating type* with one affected and one normal hearing offspring. One of these families had a dominant *GJB2* mutation, p.R75Q, in one partner and the affected offspring. This dominant mutation p.R75Q was observed for the first time in this study from India with non-syndromic presentation [11].

Interestingly, 11/60 DXD families (18.33%) had no offspring and hence could not be classically categorized as complementary/ non-complementary mating. This group too demonstrated a high frequency of *GJB2* mutations. In 6 of these families one of the mates carried *GJB2/GJB6* mutations, both novel and known, in homozygous or heterozygous condition. In one particular childless DXD family, the husband was a p.W24X carrier while the wife was W24X homozygous. Further analysis of these 11 families for deafness associated infertility genes such as *FOXI1*, *CATSPER2* and *STRC* could possibly throw more light on the etiology of this phenotype.

### DFNB1 dynamics in DXN families

**Fig. 9:**
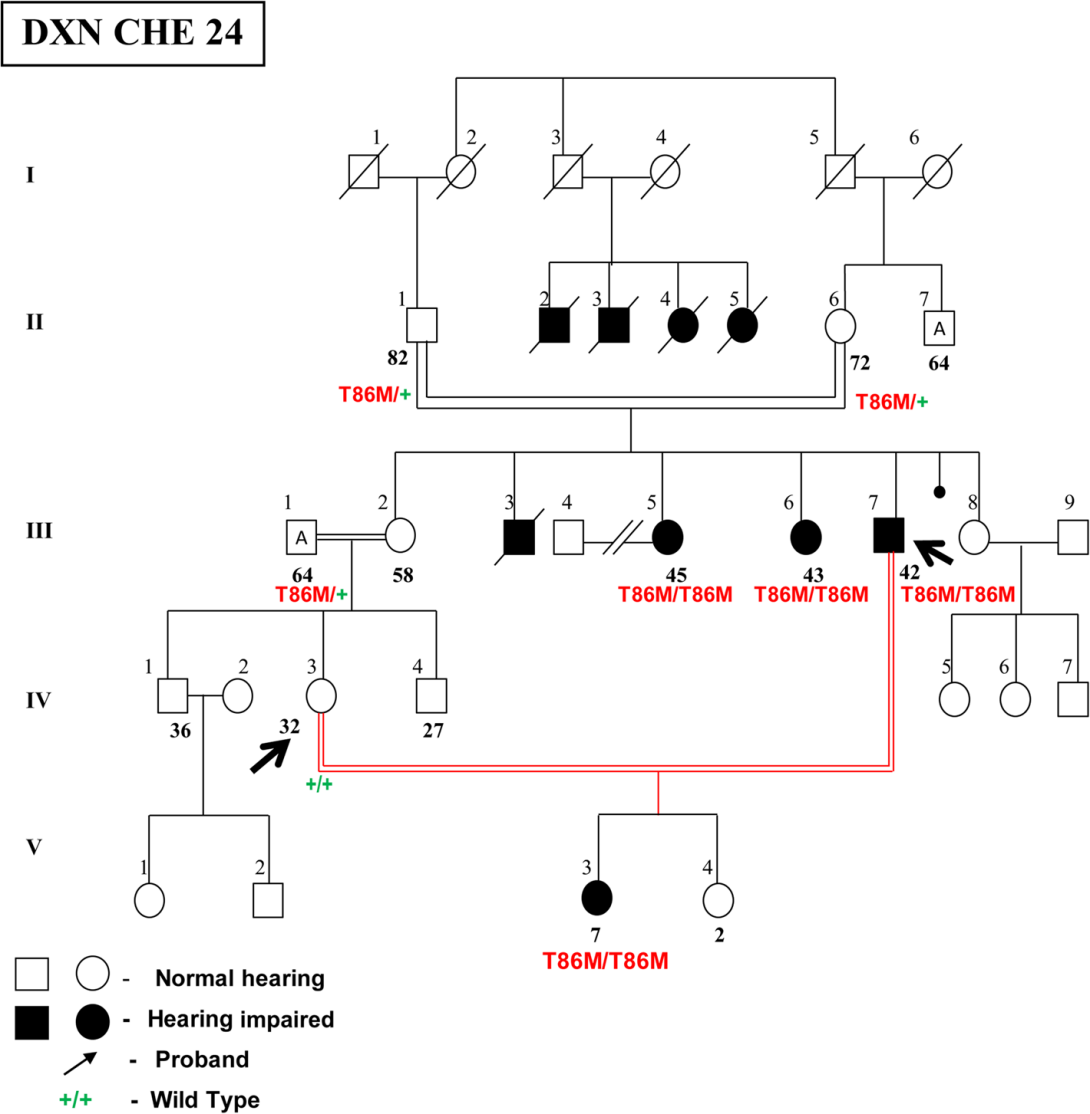
Pedigree of DXN CHE 24 family showing p.T86 M, a rare *GJB2* mutation in three consecutive generations owing to consanguinity

In our other study group of 46 DXN mating, we observed a higher rate of consanguinity (39.13%) in their marriages compared to their parental consanguinity (32.61%). Nearly 50% of the consanguineous DXN families had *GJB2* mutations. Once again, p.W24X mutation was the most common mutation in this subgroup also, with one-third of the families having this mutation in homozygous or heterozygous condition. More than 60% of these consanguineous families have affected offspring and p.W24X mutation is implicated in 60% of them. The same factor has perhaps resulted in the surfacing of a rare pathogenic variant like p.T86 M (observed for the first time in India), persistently in two consecutive generations as a result of continuous inbreeding for three generations (Fig. 9). Our findings present consanguinity as an important and additional dimension to assortative mating contributing to hearing impairment in the Indian subcontinent. It also further reiterates that consanguinity factor along with genetic drift plays an important role in the survival and initial phenotypic expression of such rare *GJB2* mutations.

Considering the non-consanguineous DXN mating families, only 7% were implicated with DFNB1 mutations. One-fourth of these families had affected offspring. Nearly 40% of the affected mates did have p.W24X mutation in homozygous or heterozygous condition. The presence of this mutation in this subgroup once again reiterates the high prevalence as well as high carrier rate of p.W24X mutation in south Indian population.

The overall allele frequency for DFNB1 mutations (*GJB2* and *GJB6* mutations) among the affected members in both the subgroups of assortatively mating was 35.67%while the carrier frequency for same among the affected members was 5.8%. The carrier frequency in the normal hearing partners of the DXN subgroup was 9.30% while among the normal hearing control group was 2.42%.

### Role of novel variants in DFNB1 loci in assortative mating

Four novel variants, p.E42D (in *GJB2* gene), p.Q57R, p.E101Q, p.R104H (in *GJB6* gene) were identified in this study. This is the first study from Indian subcontinent reporting novel variants in the coding region of *GJB6* gene. In silico analysis of these variants using popular tools such as SIFT and PolyPhen2 revealed that p.Q57R and p.R104H may affect or damage the structure and functioning of Cx30 protein coded by *GJB6*, but p.E101Q in *GJB6* and p.E42D in the *GJB2* gene are tolerable or benign to the integrity of the protein structure. However, p.E42D mutation was observed in both homozygous and heterozygous conditions in two unrelated HI individuals from different geographic and linguistic regions and with variable phenotypes in the family members ranging from mild to profound, conductive, sensorineural and mixed type of hearing losses. The individuals with other three *GJB6* novel variants in heterozygous condition have also shown profound SNHL phenotype. These variants, perhaps contribute, in association with mutations in either unrelated or yet-to-be determined loci through unknown interactive pathways, for the observed phenotype. Further investigation of these samples through whole exome sequencing and functional analysis could perhaps throw more light into their mode of action.

We calculated the allele frequency of DFNB1 pathogenic mutations, including the novel variants, in two generations of our study group, the assortative mating partners forming one generation and their offspring forming the next generation. Only families in both the subgroups that had offspring were included. The DFNB1 allele frequencies for DXD mates and their offspring were 36.98 and 38.67%, respectively and for the DXN mates and their offspring are 22.84 and 24.38%, respectively. There was a 4.6% increase in the subsequent generation in the DXD families, while a 6.75% increase in the DXN families, which demonstrates the role of assortative mating along with consanguinity in the increase of DFNB1 mutations in consecutive generations. This is perhaps the first study in the world to test real-time, the hypothesis proposed by Nance et al. in 2000 (intense phenotypic assortative mating mechanism can double the frequency of the commonest forms of recessive deafness [DFNB1]) on assortative mating HI parental generation and their offspring.

Human populations are shaped not only by the usual forces of natural selection like famine, disease or climate but also through genetic variations. A new force is emerging with surprising implications wherein people themselves have started shaping their own evolution. This new force is the human culture, broadly defined as any learned behavior including technology. Assortative mating among the deaf is one such cultural force, which along with consanguinity can have a profound influence on genetic variations, which in turn can lead to an evolutionary change in times to come. From our study, several changing trends are already noticeable at various levels that include decline in family size, deviation from previously practiced endogamous caste limitations and attitudinal changes about hearing impairment as a disability. If the preference for a deaf to marry a deaf becomes a norm in future with the advent of rampant technological advancements that empower the deaf to be better educated and economically independent, the impact on the auditory gene pool/ phenotype has several outcomes like:

1. Increase in DFNB1 mutation frequencies from existing ~ 35% as observed in our study to several folds, both in the deaf and in the normal population.
2. Deafness being a heterogeneous disorder, the remaining ~ 65% of unresolved group of gene mutations (known and unknown) will also contribute further to the gene pool and alter this equation.
3. As the genetic testing picks up gradually with cost-effective rapid multi-gene search in terms of whole genome, the knowledge may empower the prospective mates to choose partners of complementary type. This relaxed selection would again lead to simultaneous carrier status for a number of rare genes in normal hearing offspring of these mates. These normal hearing offspring may, in future, even develop variable levels of hearing losses as the proteins associated with mechanism of hearing tend to express variably.
4. Throwing newer combinations of unlinked multi-genic interactions and producing a deaf phenotype, confounding the research on the mechanism of hearing loss, there by complicating the path of unraveling the mystery of hearing.

## Conclusion

This is the first study from an Indian subcontinent reporting novel variants p.Q57R, p.E101Q, p.R104H in the coding region of *GJB6* gene. This is also the first study in the world to test real-time, the hypothesis proposed by Nance et al. in 2000 (intense phenotypic assortative mating mechanism can double the frequency of the commonest forms of recessive deafness [DFNB1]) in assortative mating HI parental generation and their offspring. The DFNB1 allele frequencies for DXD mates and their offspring were 36.98 and 38.67%, respectively and for the DXN mates and their offspring are 22.84 and 24.38%, respectively. There was a 4.6% increase in the subsequent generation in the DXD families, while a 6.75% increase in the DXN families, which demonstrated the role of assortative mating along with consanguinity in the increase of DFNB1 mutations in consecutive generations. This study has revealed that assortative mating among the deaf may well be a cultural force, which along with consanguinity can have a profound influence on genetic variations, which in turn can lead to an evolutionary change in times to come.

## Abbreviations

Cx26: Connexin 26
Cx30: Connexin 30
DFNB: Deafness, autosomal recessive
DXD: Deaf marrying deaf
DXN: Deaf marrying normal hearing
EC1: Extracellular loop 1
*GJB2*: Gap Junction *beta* 2
*GJB6*: Gap Junction *beta* 6
HI: Hearing Impaired
IC2: Intracellular domain 2
NSHL: Non-syndromic hearing loss
PCR: Polymerase Chain Reaction
SNHL: Sensory neural hearing loss
